# Maternal and Newborn Factors Associated with Meconium Metal Concentrations: A Cross-Sectional Study

**DOI:** 10.3390/toxics14020163

**Published:** 2026-02-10

**Authors:** Bianka Mimica, Ajka Pribisalic, Zlatka Knezovic, Nina Knezovic, Davorka Sutlovic

**Affiliations:** 1 Department of Gynecology and Obstetrics, University Hospital Centre Split, 21000 Split, Croatia; bmimica@kbsplit.hr; 2 Faculty of Health Sciences, University of Split, 21000 Split, Croatiadsutlovic@fzz.unist.hr (D.S.); 3 Teaching Institute for Public Health, Split-Dalmatia County, 21000 Split, Croatia; 4 Department of Applied Pharmacy, School of Medicine, University of Split, 21000 Split, Croatia

**Keywords:** meconium, newborns, essential elements, toxic metals, maternal diet, lifestyle

## Abstract

Prenatal exposure to essential and toxic metals may influence fetal development and birth outcomes. Meconium represents a valuable biomarker of cumulative intrauterine exposure; however, data linking maternal lifestyle and diet to meconium metal concentrations remain limited. This study included 152 mother–newborn pairs at the University Hospital Center Split. Meconium samples were analyzed for essential metals (Mn, Zn, Fe, Cu) and toxic metals (Hg, Pb, Cd, Ni, Cr) using atomic absorption spectrometry. Maternal and newborn characteristics were collected via questionnaires and medical records. Associations between maternal factors and metal concentrations were assessed using multivariable regression, and inter-metal correlations were evaluated with Spearman’s rank correlation. The correlation matrix indicates positive correlations among essential metals, particularly between Fe and Cu (r_s_ = 0.523), whereas toxic metals show mixed correlation patterns. Maternal factors were associated with several metal concentrations: zinc was positively associated with the newborn ponderal index; greater gestational weight gain and longer gestation were associated with lower iron concentrations; frequent fruit or grain consumption was associated with lower copper concentrations; frequent milk/dairy intake was associated with lower mercury; and fish consumption was associated with higher mercury and manganese. Rural residence and lower smoking intensity were associated with lower lead concentrations, while higher pre-pregnancy body mass index and frequent maternal smoking were associated with increased cadmium. No significant associations were observed for nickel or chromium. These findings highlight the influence of maternal diet, lifestyle, and environmental factors on fetal metal exposure, underscoring the need for monitoring, food safety control, and targeted education during pregnancy.

## 1. Introduction

Concentrations of metals in the soil, water, and atmosphere have a direct impact on environmental quality and the food chain, which consequently has a significant effect on human health [1].

Prenatal exposure to environmental pollutants, including toxic metals, represents a significant public health problem due to its potential impact on fetal development and long-term effects on growth and development. Environmental metals can cross the placental barrier and accumulate in the developing fetus [2,3,4]. Scientists have shown that toxic metals such as lead, arsenic, cadmium, and mercury negatively affect child growth, both in utero and postnatally [5]. Exposure to toxic metals has also been linked to low birth weight in newborns [6].

Many scientists have attempted to analyze how metals affect pregnancy outcomes [7]. Biomonitoring has become the method of choice for assessing exposure to various environmental factors, including metals [8,9]. Studies have been conducted to predict exposure to various metals by analyzing available biological samples [10,11,12]. Asharp et al. in their study, which included 847 women, measured metal concentrations during pregnancy in blood and urine samples [13]. Their study aimed to examine the predictive efficacy of metal biomarkers in urine and blood in relation to birth outcomes, specifically gestational age, and fetal growth outcomes. Their results suggest that, although with some limitations and the need for validation, measuring metals in urine or blood may be an equally good approach for assessing exposure to metals as a mixture.

Non-invasive biological matrices, such as meconium, have been shown to reliably reflect cumulative in utero exposure to environmental metals, particularly during the second and third trimesters of pregnancy [14,15,16]. Maternal blood or urine typically reflects short-term exposure, whereas meconium provides insight into long-term fetal exposure. Its non-invasive and simple collection makes it an ideal biomarker of prenatal metal exposure [17,18]. Various studies have shown that metal concentrations in meconium are often higher than those measured in maternal blood or umbilical cord samples, supporting its value for assessing cumulative exposure [19].

The metals selected for evaluation in this study were chosen based on their environmental prevalence, dietary exposure pathways, and documented relevance for prenatal and early-life health. Toxic metals such as lead, mercury, cadmium, nickel, and chromium are widely distributed in the environment due to industrial activity, traffic emissions, and contamination of soil and water, and are commonly detected in food products [20,21,22,23,24]. These metals can cross the placental barrier and accumulate in fetal tissues, where they may interfere with growth, neurodevelopment, and metabolic processes [2,3,4,5,6]. In addition, essential trace elements including iron, copper, manganese, and zinc were included due to their critical roles in fetal growth and development. Although required for normal physiological function, excessive or imbalanced exposure to these metals may also exert toxic effects, particularly during sensitive developmental periods [17,25,26]. Assessing both toxic and essential metals allows for a more comprehensive evaluation of prenatal exposure, potential interactions between metals, and their combined influence on metal accumulation in meconium.

Exposure to a single metal or a single factor does not provide sufficient answers to the final outcomes, i.e., concentrations in biological samples. Therefore, it is necessary to monitor exposure to several different metals, as well as parameters related to the mother and to link these factors with pregnancy outcomes.

Many factors affect the concentrations of metals in meconium. The data were categorized based on sociodemographic parameters pertaining to the mother’s habits, traits, and diet in order to determine the most significant contributions. The available results are frequently conflicting due to the inclusion of various parameters, so it is crucial to modify the analysis techniques. Multivariate statistical techniques enable the simultaneous evaluation of several variables that may have antagonistic or synergistic effects on metal accumulation. A deeper understanding of the intricate connections between biological, sociodemographic, and environmental factors is possible with this method.

Thus, the purpose of this study was to determine, by multivariate analysis, which factors mostly affect the levels of specific harmful (lead, mercury, cadmium, nickel, and chromium) and necessary (iron, copper, manganese, and zinc) metals in newborns’ meconium. The findings may advance knowledge of prenatal metal exposure and aid in the development of preventative measures meant to safeguard the health of expectant mothers and their unborn children.

## 2. Materials and Methods

### 2.1. Respondents

The study population consisted of adult mothers and their healthy newborns enrolled as mother–newborn pairs. Recruitment was conducted at the University Hospital Center Split, Department of Gynecology and Obstetrics, between January and May 2011. Mothers were approached immediately after delivery during their postpartum hospital stay and were invited to participate voluntarily in the study. The study was designed as a prospective observational investigation. Data collection included standardized birth measurements and the administration of structured questionnaires to participating mothers. The questionnaire captured detailed information on maternal characteristics, maternal lifestyle factors, and dietary habits during pregnancy. The dietary questionnaire was specifically developed for this study to assess habitual dietary patterns during pregnancy and has not been formally validated against more detailed dietary assessment methods.

In early 2025, one co-author supplemented the dataset with retrospective hospital records, adding variables including place of residence, gestational age, and parity. All participants provided written informed consent before inclusion. The study received ethical approval from the Ethics Committee of the Faculty of Medicine, University of Split (Class: 003-08/10-03/0011; Registration No.: 2181-198-04-10-0002) on 4 May 2010.

### 2.2. Samples

Meconium samples were collected within the first 24 h after birth. They were removed from the newborn’s diaper using sterile, disposable plastic spoons and placed in sterile plastic containers to minimize the risk of external metal contamination. Samples were stored at −20 °C until analysis. Each sample was labeled with a unique identification number corresponding to the questionnaire completed by the mother at the same time. Prior to analysis, the samples were dried to a constant weight at 40 °C, and all results were expressed on a dry weight basis. Approximately 1 g of well-homogenized meconium sample was directly weighed into Teflon digestion tubes for the wet digestion procedure. The digestion was performed using an automated microwave digestion system Mars 5 (CEM Corporation, Matthews, USA) with a mixture of concentrated nitric acid (HNO_3_), hydrochloric acid (HCl), and hydrogen peroxide (H_2_O_2_). All reagents used were of suprapure grade (Merck, Darmstadt, Germany).

### 2.3. Metal Concentration Detection

The quantitative determination of essential metals: manganese (Mn), zinc (Zn), iron (Fe) and copper (Cu) was performed using an atomic absorption spectrometer (AAS) on a Vario 6 atomic absorption spectrometer (Analytik Jena, Jena, Germany) operated in flame mode. The limits of detection (LOD) were calculated from standard deviations of the blanks and were 0.01 mgL^−1^ for Zn and Mn, while for Fe and Cu LOD was 0.05 mgL^−1^. Sample preparation and processing procedures have been described in detail in a previously published study [17].

The quantitative determination of toxic metals: lead (Pb), cadmium (Cd), nickel (Ni) and chromium (Cr) was performed using graphite furnace atomic absorption spectrometry (GF-AAS) on the same Vario 6 spectrometer (Analytik Jena, Jena, Germany) equipped with deuterium background correction. Mercury (Hg) concentrations were determined using an Advanced Mercury Analyser AMA 254 (Altec, Prague, Czech Republic), by direct analysis of a homogenised sample without previous preparation.

The limits of detection (LOD), calculated from the standard deviation of blank measurements, were 1.0 µgL^−1^ for Pb, Cr, Ni; 0,3 µgL^−1^ for Hg, and 0.1 µgL^−1^ for Cd. The accuracy of the analytical methods was validated using the certified reference material Seronorm Trace Elements Urine L-2 (lot no. 210705; Sero AS, Hvalstad, Norway). The obtained results showed good agreement with the certified values, with recoveries of 94.84% for Pb; 95.92% for Cd; 98.04% for Hg; 109.81% for Cr and 109.08% for Ni.

During measurements, method accuracy was additionally verified by analyzing standard solutions of known concentrations processed as samples. All standards were prepared from certified stock solutions (1000 ± 2 mg/L, Suprapur grade) obtained from Merck (Darmstadt, Germany).

### 2.4. Data

Data on maternal and newborn characteristics were collected from hospital medical records and structured questionnaires completed by mothers. The collected information included anthropometric, demographic, and lifestyle variables.

Maternal anthropometric data, including height, pre-pregnancy weight, and weight at delivery, were self-reported by mothers via questionnaire. Based on these measurements, maternal pre-pregnancy BMI and BMI at delivery were calculated. Newborn anthropometric measurements, including birth length and birth weight, were obtained from medical records. Based on these data, the ponderal index, an indicator of newborn adiposity, was calculated using the formula: newborn birth weight in grams × 100/cubed newborn birth length in centimeters [27].

Additional variables included maternal age, place of residence, smoking status, and dietary habits. Dietary information comprised the frequency of consumption of meat, fish, dairy products, fruits, vegetables, and grains, as well as intake of coffee, tea, and alcoholic beverages (including hard liquor and wine). The gestational age at delivery, expressed in completed weeks of gestation, was recorded for each participant.

### 2.5. Statistical Analysis

The Shapiro–Wilk test was applied to verify data normality, given the moderate sample size (*N* = 152) and the test’s suitability for smaller datasets (*N* < 200). Median values with interquartile ranges and total ranges were calculated as descriptive measures. Associations between the measured concentrations of essential metals were examined using Spearman’s rank correlation coefficients.

Associations between maternal and newborn characteristics and metal concentrations in meconium were examined using multivariable linear regression analysis. Separate regression models were constructed for each metal (Mn, Zn, Fe, Cu, Hg, Pb, Cd, Ni, and Cr), with all independent variables entered simultaneously into each model. The independent variables included maternal age, pre-pregnancy body mass index (BMI), gestational weight gain, gestational age at delivery, newborn ponderal index, place of residence: urban vs. rural [28], maternal smoking status, and maternal dietary intake during pregnancy.

In addition, a multivariable linear regression model was fitted with newborn ponderal index as the dependent variable to examine associations with maternal and lifestyle factors, including maternal age, pre-pregnancy BMI, gestational weight gain, gestational age at delivery, dietary intake, smoking status, and place of residence.

Multicollinearity among independent variables was assessed using variance inflation factors (VIFs), with all values remaining below commonly accepted thresholds, indicating no evidence of problematic multicollinearity. Alcohol consumption was not included in the models due to the very low number of cases, which would have resulted in unstable estimates and reduced model reliability. Regression results are reported as unstandardized regression coefficients (B) with 95% confidence intervals (CI) and corresponding *p*-values. For clarity, B coefficients and confidence intervals are presented only for statistically significant associations (*p* < 0.05), whereas *p*-values alone are shown for non-significant associations.

All statistical analyses were performed using IBM SPSS version 21. Statistical significance was set at *p* < 0.050.

## 3. Results and Discussion

### 3.1. Participant Information

Mother–newborn pairs were excluded from the analysis if questionnaire data were incomplete or if an insufficient amount of meconium was available for analysis. After applying these exclusion criteria, a total of 152 mother–newborn pairs were included in the final study sample.

The gestational age at delivery ranged from 36 to 42 weeks. The minimum recorded birth weight was 1780 g, and the maximum was 4820 g. The mean birth weight of newborns was 3576.09 g (SD = 37.51). Birth length ranged from 47 to 55 cm, with a median value of 51 cm. Table 1 summarizes maternal and newborn characteristics, including maternal age, pre-pregnancy BMI (BMI-1), BMI at delivery (BMI-2), ponderal index as an indicator of newborn nutrition status, and gestational age.

Of the total number of mothers, 75.70% were non-smokers, 18.40% smoked up to 10 cigarettes per day, and 5.90% smoked 10–20 cigarettes per day. Regarding place of residence, 72.4% of participants lived in urban areas (including urban, small towns, and industrial areas), while 27.6% resided in rural areas.

### 3.2. Metals Concentration in Meconium Samples

Meconium samples were analyzed for two categories of metals: essential metals (Mn, Zn, Fe, and Cu) and toxic metals (Hg, Pb, Cd, Ni, and Cr). Table 2 presents the concentrations of all analyzed metals. The concentrations of essential metals are expressed in mg of metal per g of dry meconium sample, whereas toxic metal concentrations are expressed in ng of metal per g of dry meconium (Table 2). For Pb, Cd, and Ni, the minimum measured concentrations were below the LOD values.

**Figure 1:**
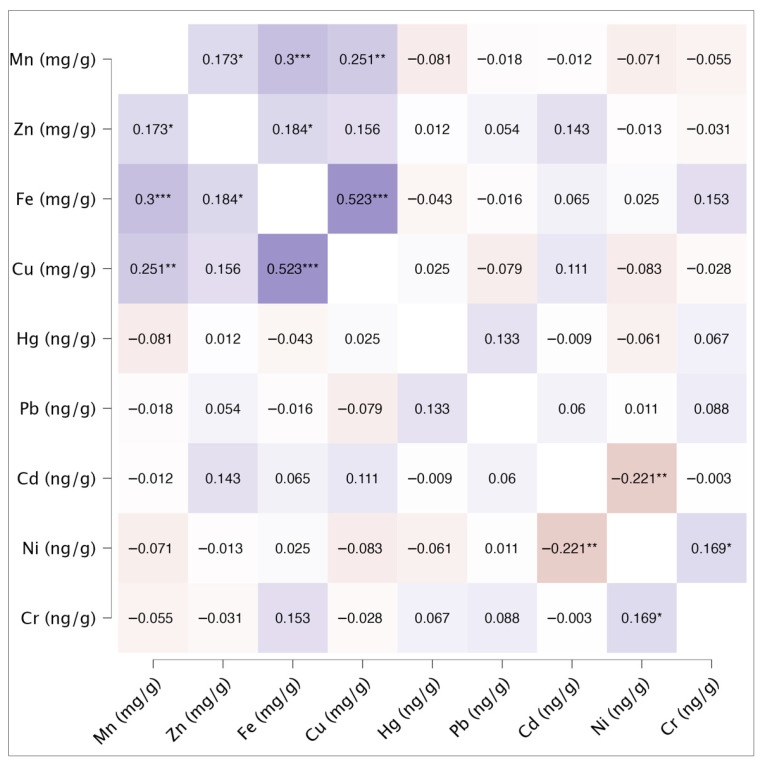
Spearman correlation heatmap of metal meconium concentrations (*N* = 152). Purple shades show positive correlations (darker means stronger), while terracotta shades show negative correlations (darker means stronger). Significance level was denoted with * (*p  <  0.05, ** *p*  <  0.01, *** *p*  <  0.001).

Figure 1 presents Spearman’s rank correlation coefficients among the analyzed metals. Most metal pairs exhibited significant positive correlations, with Fe and Cu showing the strongest association (r_s_: 0.523). Within the group of toxic metals, only Cr and Ni demonstrated a statistically significant but weak correlation (r_s_ = 0.169), which is notably lower than the correlation reported by Michael et al. (r_s_ = 0.77) [29]. A statistically significant negative correlation coefficient was observed between Ni and Cd (r_s_: −0.221). Additionally, weaker correlations were observed between metals from different groups, including Zn–Ni (r_s_ = −0.13) and Fe–Cr (r_s_ = 0.153), none of which reached statistical significance.

Kou et al. investigated the effects of maternal heavy metal exposure during pregnancy on newborns’ neurological development by analyzing maternal urine samples collected during the first trimester [30]. Michael et al. also examined urine samples [29]. Both studies reported only positive correlations among the metals under investigation. In the Kou et al. study, Spearman’s correlation coefficients (r_s_) between heavy metals ranged from 0.03 to 0.24, with the strongest association observed between Cd and Pb (r_s_ = 0.24) [30]. Similarly, Michael et al. reported a correlation of 0.25 for the same elements [29]. Shin et al. reported a somewhat lower correlation (r_s_ = 0.12) for Cd and Pb, based on maternal blood samples collected between gestational weeks 6 and 32 [31]. In our study, the correlation between Cd and Pb was markedly lower (r_s_ = 0.06), whereas the correlation between Ni and Cr was statistically significant (r_s_ = 0.169).

Given the substantial differences in matrices used to assess metal content, it is essential to evaluate actual newborn exposure accurately. Comparisons of heavy metal measurements across various matrices, including maternal urine, neonatal urine, and cord blood, have shown inconsistent results [32]. Therefore, meconium analysis, in contrast to blood or urine analysis, provides valuable information on cumulative exposure and, more specifically, reflects the newborn’s integrated heavy metal burden over gestation.

### 3.3. Multivariate Analysis

Multivariable linear regression analyses were performed to examine the associations between maternal and newborn characteristics (including anthropometric characteristics, maternal diet, and lifestyle habits) and meconium metal concentrations (Table 3). All independent variables were entered simultaneously into each model.

Although fish is not considered the primary source of Mn, our results showed that Mn concentrations in meconium were lower in newborns of mothers who did not consume fish compared to those who consumed fish (B = −7.83, *p* = 0.034). Cereals are an important source of Mn [33]; however, in our study, cereal consumption was not statistically significantly associated with Mn levels. Zinc concentrations in meconium were positively associated with the ponderal index of the newborn, such that a higher ponderal index corresponded to increased Zn concentrations (B = 188.3, *p* = 0.010).

Several dietary factors were significantly associated with copper concentrations in meconium. Occasional fruit consumption, compared with frequent consumption, was positively associated with Cu levels (B = 16.38, *p* = 0.013). This finding may be explained by previous research indicating that elevated copper concentrations in certain fruits can result from fungicide use and soil contamination [34,35]. For example, Kos et al. reported the highest copper concentration in stone fruit soils [34]. Additionally, infrequent or occasional grain consumption was associated with higher Cu concentrations in meconium (B = 12.64, *p* = 0.034).

An earlier study reported higher Mn, Zn, and Cu concentrations and lower Fe concentrations in the meconium of term compared with preterm newborns [36]. In our study, iron concentrations in meconium were inversely associated with gestational weight gain (B = −1.46, *p* = 0.006) and gestational age (B = −4.32, *p* = 0.042), with lower Fe concentrations observed at longer gestational durations.

Importantly, iron concentration in meconium is thought to reflect regulated fetal iron transfer and excretory dynamics rather than iron storage or iron status per se. Previous work suggests that fetal iron handling, particularly in the third trimester, is tightly regulated and non-linear, with meconium reflecting iron flux rather than biologically available iron reserves. Accordingly, lower meconium iron concentrations at longer gestational ages may indicate more efficient intrauterine regulation and utilization of iron rather than reduced fetal iron supply. In healthy term newborns, meconium iron concentration is therefore not considered a strong or clinically necessary marker of fetal iron status [37].

It is also important to note that the absorption, metabolism and excretion of essential metals are complex and non-linear processes influenced by interactions with other essential and toxic elements [38]. The presence of certain element combinations may either enhance or inhibit absorption and utilization [38], further supporting the interpretation that changes in meconium iron concentration reflect regulated physiological processes rather than linear accumulation or depletion. Nevertheless, these findings should be interpreted as associative and hypothesis-generating, highlighting the complexity of fetal iron metabolism rather than a linear process of iron loss or reabsorption.

Since our previous study provided a detailed analysis of the impact of essential metals on birth outcomes [17], the following sections focus primarily on toxic metals. Numerous studies have documented the adverse health effects of maternal exposure to heavy metals during pregnancy [30,39]. In particular, in utero and postnatal growth of children has been shown to be negatively affected by toxic metals such as arsenic, mercury, lead, and cadmium [5,40].

In light of these statements, we consider it important to further explore potential associations between the maternal diet and lifestyle during pregnancy and metal concentrations in meconium. Understanding these relationships may provide insights into strategies to reduce fetal exposure to toxic metals and mitigate their potential health effects. Our study demonstrated that several examined maternal parameters significantly influenced the resulting mercury content in meconium.

Meconium Hg concentrations were strongly associated with specific dietary habits. The absence of maternal fish consumption was associated with significantly lower Hg concentrations (B = −52.73, *p* < 0.001), compared to fish consumers. Conversely, infrequent consumption of milk and dairy products was associated with higher Hg concentrations (B = 41.46, *p* < 0.001) relative to frequent consumers.

Increased Hg levels associated with higher fish consumption have also been reported by other investigators [41]. Hg levels in maternal blood/serum, placenta, and cord blood/serum were observed to increase proportionally with increased fish intake, with the highest levels reported in coastal populations, likely due to greater availability and consumption of fish [41]. In our study, 70% of mothers reported frequent fish consumption. Questionnaire data indicated that mothers residing on islands consumed approximately twice as much fish as mothers from rural mainland areas. According to Knezovic et al., elevated Hg concentrations may also result from mercury deposition from industrial facilities [42]. In addition, Trdin et al., using meconium analysis, reported a significant correlation between Hg content and seafood consumption [43]. In contrast to Sekovanić et al. [41], our study did not observe an association between higher Hg concentrations and the presence of maternal dental amalgam fillings.

Place of residence and maternal smoking were significantly associated with lead concentrations in meconium. Rural residence was associated with substantially lower Pb concentrations compared with urban residence (B = −430.72, *p* < 0.001), which is particularly notable given the median Pb concentration of 398.76 ng/g. According to Knezovic et al.’s study, which thoroughly investigated the environmental pollution caused by heavy metals and meconium bioaccumulation, the influence of the place of residence is definitely linked to pollution from industrial plants, road traffic sources, and landfills in the studied area [44]. In their analysis of 151 meconium samples, Pb was detected in 89.4% and Cd in 94.0% of samples. Other studies have also highlighted the effect of residence; for example, research in Pakistan found that mothers living in industrial cities with steel mills had the highest Pb levels, emphasizing the importance of industrial waste management to prevent population exposure, particularly in pregnant women [19]. In addition, non-smoking mothers exhibited lower Pb concentrations compared with mothers smoking 10–20 cigarettes per day (B = −359.03, *p* = 0.034). Maternal smoking during pregnancy has been associated with increased Pb concentrations [45,46], increased Cd levels in the maternal, placental and fetal spaces, and reduced Fe in the placenta [46].

Given that newborn brain development is negatively affected by cadmium exposure [30], it is critical to identify factors contributing to elevated levels of this metal. In our study, pre-pregnancy maternal BMI and smoking status were significant predictors of cadmium concentrations. Higher pre-pregnancy BMI was associated with increased Cd concentrations (B = 0.59, 95% CI 0.21–0.97, *p* = 0.002). Compared with mothers smoking 10–20 cigarettes per day, both non-smokers (B = −8.19, 95% CI −13.39 to −3.00, *p* = 0.002) and mothers smoking up to 10 cigarettes per day (B = −8.83, 95% CI −14.48 to −3.17, *p* = 0.002) had significantly lower Cd concentrations. Previous studies have reported that cadmium levels in the placentas of smokers can be 10–20 times higher than corresponding maternal blood concentrations, which is attributed to smoking [47]. In non-smokers, placental Cd levels were considerably lower, and differences were thought to reflect dietary influences rather than tobacco exposure [45]. Consistent with this, our study found that infrequent grain consumption was associated with higher Cd concentrations in meconium (B = 3.52, 95% CI 0.69–6.36, *p* = 0.015) compared with frequent consumption.

No statistically significant associations were observed between maternal or newborn characteristics and meconium concentrations of nickel or chromium.

Maternal age and the consumption of meat, vegetables, tea, and coffee appeared to have little or no impact on the concentrations of the analyzed metals. Among all factors examined, place of residence exerted the strongest influence on Pb levels. Recommendations to increase the intake of foods or supplements with antioxidant properties have been proposed as a potential strategy to mitigate the effects of dietary and environmental exposure to heavy metals [48].

Several approaches have been used to evaluate the effects of metal concentrations on birth outcomes. Newborn anthropometric measurements, including weight, length, and head circumference, can be directly assessed, or indices derived from the scientific literature, such as weight-for-gestational-age indices, may be utilized [12,49]. For example, a study of 125 mother–newborn pairs examined metal concentrations in maternal blood in relation to delivery outcomes, suggesting that prenatal exposure to metals may influence birth outcomes [31].

In our study, the Ponderal index was used to assess newborn characteristics, providing a more comprehensive measure to explain variations in birth weight and length. Additional multivariable linear regression analysis was conducted to examine the associations between maternal characteristics, lifestyle factors, and dietary intake during pregnancy and the newborn ponderal index. The analysis included 152 mother–newborn pairs with complete data. Among continuous predictors, gestational age was positively associated with ponderal index (B = 0.058, 95% CI 0.026–0.090, *p* < 0.001), indicating higher ponderal index values with increasing gestational duration. Gestational weight gain was also significantly associated with ponderal index (B = 0.011, 95% CI 0.002–0.019, *p* = 0.011). Maternal age and pre-pregnancy BMI were not significantly associated with ponderal index. None of the categorical predictors, including maternal dietary intake (fish, meat, fruit, vegetables, grains, milk and dairy products, tea, or coffee), maternal smoking status, or place of residence during pregnancy, showed statistically significant associations with ponderal index.

Pregnancy outcomes, as previously discussed, may be influenced by metal concentrations, which in turn are closely related to placental function [50,51]. However, some studies that examined only placental metal concentration did not demonstrate significant associations, which has been attributed to low levels of exposure [51]. In contrast, studies analyzed maternal serum during the second trimester have provided epidemiological evidence supporting a link between maternal metal exposure and newborn birth outcomes [52].

Considering the present findings, the population most vulnerable to elevated toxic metal exposure appears to be those residing in industrial, highly urbanized areas, as well as those living in island/coastal areas. Our results indicate that women from urban regions exhibit significantly higher concentrations of Hg and Pb in meconium compared with mothers from rural areas. Among the factors examined, fish consumption and place of residence had a greater influence on Hg and Pb levels than maternal smoking or other lifestyle variables. It is noteworthy that 75% of the 152 mothers in our cohort were non-smokers, highlighting the need for caution when interpreting the effects of smoking on heavy metal concentrations.

Furthermore, we emphasize that the potential impact of dietary exposure to heavy metals should not be underestimated, even though our results did not demonstrate strong associations between metal concentrations and the consumption of specific food items. Bioaccumulation of heavy metals through the food chain can have a significant influence, particularly in urban and industrial environments, potentially leading to elevated metal levels in food products. The findings of Knezovic et al., which analyzed Hg, Pb, and Cd concentrations in 3528 food samples, including fruits, vegetables, seafood, meat, cereals, milk, and dairy products, are particularly concerning [53]. According to their findings, 61% of the samples contained Hg, 83% contained Pb, and 78% contained Cd, with more than 30% of the samples exceeding 75% of the maximum allowable limits. Comparable findings were reported by Garofalo L. et al., who monitored a large number of seafood samples over a ten-year period and observed elevated mercury concentrations in marine fish [54]. Similarly, Eissa F. et al. analyzed data from the European Community Rapid Alert System for Food and Feed (RASFF), which documents market withdrawals due to excessive levels of heavy metals [55]. Their analysis, covering a 23-year period, revealed that the highest proportions of notifications were associated with mercury (36.6%), cadmium (25.1%) and lead (14.1%).

These data suggest that different dietary patterns, including adherence to a healthy Mediterranean diet, may not always ensure reduced metal intake and mitigate their impact on birth outcomes. Indeed, some studies indicate that adherence to the Mediterranean dietary pattern may not protect against cadmium exposure [56]. It should be noted, however, that our study has limitations in this regard, as we did not specifically investigate adherence to the Mediterranean diet.

## 4. Conclusions

In conclusion, our findings underscore the importance of continuous monitoring of food for heavy metal contamination and the implementation of programs aimed at reducing environmental emissions of toxic metals. To strengthen these conclusions, further research is needed to assess the cumulative effects of a broader spectrum of metals, considering additional factors that may influence metal concentrations in biological samples. Moreover, large-scale cohort studies incorporating maternal factors, birth outcomes, and child development would provide valuable insights into the long-term effects of prenatal metal exposure and inform potential preventive strategies. Future studies should also include more comprehensive socioeconomic and lifestyle information to better account for potential confounding influences on fetal metal exposure.

## Figures and Tables

**Table 1:** Characteristics of Mothers and Newborns (*N* = 152).

	Mother’s Age	BMI-1	BMI-2	Ponderal Index	Gestation (Weeks)
Median	31.00	22.10	27.63	2.74	40.00
IQR	8.00	3.60	4.54	0.30	1.00
Minimum	19.00	16.46	19.75	1.61	36.00
Maximum	44.00	33.14	38.82	3.52	42.00

BMI-1: pre-pregnancy BMI; BMI-2: BMI at delivery.

**Table 2:** Summary of metal concentration levels (mg/g and ng/g) in meconium samples.

	Mn (mg/g)	Zn (mg/g)	Fe (mg/g)	Cu (mg/g)	Pb (ng/g)	Cd (ng/g)	Ni (ng/g)	Cr (ng/g)	Hg (ng/g)
Median	19.44	331.39	53.45	71.67	398.76	9.92	167.78	131.99	35.68
IQR	18.31	234.39	39.86	39.88	561.88	6.22	387.82	88.28	53.95
Minimum	1.93	25.30	12.81	24.06	<LOD	<LOD	<LOD	1.000	2.50
Maximum	151.60	1035.22	149.40	204.40	2423.64	78.58	2233.90	1180.05	394.69

LOD: limits of detection.

**Table 3:** Linear regression models of maternal and newborn characteristics associated with meconium metal concentrations (Mn, Zn, Fe, Cu, Hg, Pb, Cd, Ni, Cr) in 152 subjects. All independent variables were included simultaneously in each model. Each column represents a separate regression model for one metal. Cells report B (unstandardized regression coefficient), 95% CI (confidence interval), and *p* (*p*-value). For non-significant associations (*p* ≥ 0.05), only the *p*-value is shown; B and CI are reported only for statistically significant associations (*p* < 0.05).

Variables		Mn	Zn	Fe	Cu	Hg	Pb	Cd	Ni	Cr
Mother’s age	*p*	0.293	0.127	0.849	0.451	0.914	0.267	0.567	0.775	0.570
BMI pre-pregnancy	B							0.591		
	CI							0.21, 0.97		
	*p*	0.544	0.852	0.163	0.458	0.160	0.671	0.002	0.989	0.268
Weight gain	B			−1.46						
	CI		−2.49, −0.42							
	*p*	0.797	0.622	0.006	0.051	0.419	0.304	0.745	0.185	0.180
Gestation weeks	B			−4.32						
	CI		−8.49, −0.15							
	*p*	0.748	0.271	0.042	0.105	0.235	0.345	0.749	0.655	0.139
Ponderal index	B		188.3							
	CI	45.03, 331.6								
	*p*	0.065	0.010	0.397	0.554	0.758	0.572	0.105	0.659	0.167
Place of residence: rural (Ref. Urban)	B						−430.72			
	CI					−598.07, −263.38				
	*p*	0.369	0.194	0.530	0.362	0.074	<0.001	0.330	0.737	0.806
Smoking (Ref.: 10 to 20 cigarettes per day), non-smokers	B						−359.03	−8.19		
	CI						−690.67, −27.40	−13.39, −3.00		
	*p*	0.194	0.617	0.275	0.215	0.724	0.034	0.002	0.406	0.491
up to 10 cigarettes per day	B							−8.83		
	CI							−14.48, −3.17		
	*p*	0.515	0.391	0.969	0.788	0.795	0.131	0.002	0.356	0.744
**Weekly consumption of**										
Meat: No/sometimes (Ref.: Often)	*p*	0.739	0.857	0.612	0.711	0.305	0.404	0.553	0.788	0.549
Fish: No (Ref.: Yes)	B	−7.83				−52.73				
	CI	−15.05, −0.62				−75.26, −30.20				
	*p*	0.034	0.450	0.438	0.344	<0.001	0.474	0.675	0.938	0.145
Fruit: Sometimes (Ref.: Often)	B				16.38					
	CI				3.50, 29.30					
	*p*	0.487	0.223	0.072	0.013	0.795	0.133	0.708	0.678	0.311
Vegetable: Sometimes (Ref.: Often)	*p*	0.178	0.654	0.261	0.665	0.719	0.274	0.682	0.131	0.193
Grains: No/sometimes (Ref.: Often)	B				12.64			3.52		
	CI				0.98, 24.30			0.69, 6.36		
	*p*	0.169	0.140	0.954	0.034	0.529	0.916	0.015	0.350	0.648
Milk and dairy products: No/sometimes (Ref.: Often)	B					41.46				
	CI					17.22, 65.71				
	*p*	0.716	0.232	0.205	0.880	<0.001	0.677	0.315	0.632	0.882
Tea: No/sometimes (Ref.: Often)	*p*	0.303	0.064	0.499	0.475	0.606	0.602	0.719	0.050	0.971
Coffee and similar beverages: No (Ref.: Yes)	*p*	0.967	0.477	0.924	0.483	0.845	0.518	0.348	0.583	0.418

## Data Availability

Data supporting the findings of this study can be obtained from the corresponding author upon request.
